# Does Mental Health Status Influence Susceptibility to the Physiologic Effects of Air Pollution? A Population Based Study of Canadian Children

**DOI:** 10.1371/journal.pone.0168931

**Published:** 2016-12-28

**Authors:** Robert E. Dales, Sabit Cakmak

**Affiliations:** 1 Population Studies Division, Health Canada, Ottawa, Ontario, Canada; 2 School of Medicine, University of Ottawa, Ottawa, Ontario, Canada; Institute of Psychiatry, UNITED KINGDOM

## Abstract

**Background:**

Both air pollution exposure and the presence of mental illness are associated with an increased risk of physical illness.

**Objective:**

To determine whether or not children with less favourable mental health are more susceptible to pulmonary and cardiovascular effects of ambient air pollution, compared to those who are mentally healthy.

**Methods:**

We carried out a cross-sectional study of 1,883 children between the ages of 6 and 17 years of age who participated in the Canadian Health Measures population survey between 2007 and 2009. Subjects were assigned the air pollution values obtained from the National Air Pollution monitor closest to their neighborhood. Lung function, heart rate and blood pressure were stratified by indicators of mental health. The latter were ascertained by questions about feelings of happiness, a diagnosed mood disorder, and the emotional symptom subscale of the Strengths and Difficulties Questionnaire.

**Results:**

Among those who reported a mood disorder, an interquartile increase in ozone was associated with increases in systolic and diastolic pressures of 3.8 mmHg (95% CI 1.6, 5.9) and 3.0mmHg (95%CI 0.9, 5.2) respectively, and a decreases in FVC of 7.6% (95% CI 2.9, 12.3). No significant changes in these variables were observed in those who did not report a mood disorder. Among those with unfavourable emotional symptoms, ozone was associated with a 6.4% (95% CI 1.7, 11.3) increase in heart rate, a 4.1% (95%CI 1.2, 7.1) increase in systolic blood pressure, and a 6.0% (95% CI 1.4, 10.6) decrease in FEV_l_. No significant effect was seen in these variables among those with no emotional symptoms.

**Conclusions:**

In the Canadian population, children who report mood disorders or unfavourable emotional symptoms appear to be more vulnerable to the adverse physiologic effects of air pollution.

## Introduction

Exposure to ambient air pollution has been associated with an increase in biomarkers of inflammation and oxidative stress, physiologic impairment, and cardiovascular and respiratory morbidity and mortality. The existence of susceptible subgroups should be identified and taken into account when interpreting studies on the population health effects of air pollution. Setting air pollution guidelines based on observed effects in the general population may be insufficient to protect especially vulnerable subgroups. The magnitude of the observed effect of air pollution in any given study will be influenced by the proportion of vulnerable people exposed [1]. Also the most susceptible may derive the most benefit from reduction in air pollution. Identifying the especially vulnerable would be a first step in focusing prevention strategies. For example, the Air Quality Health Index communicates to the public both a summary of the air quality and preventative advice which differs between those with and without an elevated risk due to chronic lung or heart disease and the general population [2].

Susceptible subgroups have been identified. Based on modelling the expected health benefits of reducing emissions from fossil-fuel power plants, Levy et al. [3] reported disproportionate mortality reductions among African Americans, those with less than a high school education, and those with diabetes. Evidence has emerged that the socially disadvantaged, very young, very old, and those with chronic cardiopulmonary disease may bear a higher proportion of the burden of illness from air pollution than the general population [4].

The impact of air pollution on children with mental health disorders has not been addressed. We previously found that, compared to less happy adults, those who were happy had fewer significant associations between selected ambient air pollutants and selected blood pressure and lung function measurements [5]. Another study suggested that depression may increase the risk of an adverse reaction to an environmental exposure. Hicken MT et al. [6] reported an interactive effect of depression and ethnicity on the association between blood pressure and lead. Among those who were depressed, Blacks had a larger increase in blood pressure for a doubling of blood lead than did Whites. This differential effect was not seen among those who were not depressed. Apart from this empiric evidence suggesting an interactive effect, we suggest that there are multiple possible reasons why those with mental illness may be more susceptible to the adverse effects of air pollution: an augmentation of oxidative stress and inflammation, unfavourable self-care behaviours (not seeking care, not adhering to treatment, smoking), or lack of social and financial support [7–10]. The social and behavioural factors could minimize taking corrective actions to protect or improve health.

The subject of the interaction between air pollution and mental illness is highly relevant in our society given that air pollution is ubiquitous and mental illness is common and a cause of significant morbidity. A 2006 Canadian federal government report titled “The Human Face of Mental Health and Mental Illness in Canada” [11] described the large burden of illness related to mental health issues. Of those at least fifteen years of age, it was estimated that 5% met the criteria for a mood disorder and 5% for an anxiety disorder. According to this report the estimated direct economic costs which include medications, physician services, hospitalization and long-term disability, approached five billion dollars in Canada based on 1998 data. In developed countries, one-to-six percent of mental illnesses were associated with severe disability. In developing countries disability associated with mental illness was reported to be greater than for most physical illnesses [12]. Mental illness has been associated with an increase in biomarkers of inflammation [7,8,13,14], and mortality from cardiovascular and respiratory diseases, and cancer [15].

The primary objective of the present study was to determine if mental health influences susceptibility to the adverse effects of air pollution among children. We used data from the Canada Health Measures Survey to test the associations between air pollution, and lung function, heart rate and blood pressure, and stratified the results by more v. less favourable indicators of mental health.

## Research Methods

### Study Population

The target population based on national census data was Canadians between 6 and 79 years old. Exclusions, comprising a total of about 4%, were for those who lived in remote areas, institutions, reserves, and those in the armed forces. Since physiologic testing was required, clinical examination centers were necessary. Cost and logistic constraints restricted the number of to fifteen. To select the geographic location of the sites, the country was divided into 257 potential sampling sites of a minimum of 10,000 people living within 50–100 km of each other, and then these sites were grouped into five regions across Canada: British Columbia, the Prairies, Ontario, Quebec, and Atlantic provinces. Fifteen sites were selected from these regions using probability sampling based on population size. Within each site, simple random sampling of approximately 600 households was done, stratified by age group. Households were sent a letter of introduction. A trained interviewer then visited the home at a mutually agreeable time and administered a standardized questionnaire. Afterwards, a time was set up to carry out the physical measures.

Of the approximate 70% of households that provided information on the age and sex of all household members, approximately 88% completed a household questionnaire, and of these, approximately 85% attended the physical exam center. For the present study, we selected those children between six and seventeen years old who had a completed both a questionnaire and physiologic testing. To create a representative population sample, survey weights were assigned after the responding sample was determined to adjust for non-response and non-random sample selection. Standard errors and confidence intervals were calculated by bootstrapping [16].

### Research Ethics

The study was assessed by the Office of the Privacy Commissioner of Canada, and approved by the Health Canada Research Ethics Board. Participation was voluntary. For the household questionnaire, as with most StatCan surveys, implied consent was obtained. Respondents were told about the voluntary nature of the survey and chose to answer, or not answer questions as they were asked. For the clinic visit (physical measures and specimen collection), written consent was obtained from 14–79 year olds, and parents or legal guardians provided written consent for 3–13 year olds. Six to 13 year olds provided assent. Consent was recorded on both the paper documents which are kept securely at StatCan Head Office and in a secure computer assisted collection application. The Health Canada Research Ethics Board approved the consent procedures for all age groups.

### Questionnaire

The questionnaire was administered by a trained interviewer according to a standardized protocol clinic questionnaire [17].

#### Happiness

The indicator question was: ‘Would you describe yourself as being usually: … happy and interested in life… somewhat happy… somewhat unhappy… unhappy with little interest in life… so unhappy that life is not worthwhile?’

#### Mood disorder

This was indicated by a yes or no answer to the question: ‘Do you have a mood disorder such as depression, bipolar disorder, mania or dysthymia [diagnosed by a health care professional]?

#### Children’s emotional symptoms

Emotional difficulties were assessed using the Emotional Symptoms Scale which is part of the Strengths and Difficulties Questionnaire (SDQ) developed by Robert Goodman [18]. It has been widely used in many countries and in many languages [19,20] Parents were asked to respond to the following descriptions of their child based on the ‘child's behaviour over the last six months or this school year’:

1. Complains of headaches, stomach aches or sickness
2. Many worries
3. Often unhappy, depressed or tearful
4. Nervous/clingy in new situations, easily loses confidence
5. Has many fears, is easily scared

Response options were: ‘Not True’,’ Somewhat True’, or ‘Certainly True’, and scored as 0, 1, or 2 respectively. The sum of the scores for each question was used for analysis.

### Air Pollution and Weather

The Canadian Health Measures Survey used air quality data from area monitors closest to the clinical examination site. Each participant was assigned the data obtained on the day of his/her testing. 69.8% of the respondents lived relatively close to the clinic site, within approximately 11.00 km. Eleven monitors were within 10km of the study site, two others were 22 and 25km away, and 2 were 65 and 158km from the site. Meteorological data were supplied by the National Climate Data and Information Archive and air pollution data were provided by The Canadian National Air Pollution Surveillance (NAPS) network which has approximately 300 monitoring stations in about 200 communities. Particulate matter of median aerodynamic diameter less than 2.5 microns (PM_2.5_) was measured using a tapered element oscillating microbalance monitor, O_3_ was estimated using ultraviolet absorbance, and NO_2_ was quantified by chemiluminescence. For analysis, we used the maximum eight-hour average for O_3_, and the 24-hr means for PM_2.5_, and NO_2,_ temperature, humidity and barometric pressure measured on the day of the test for each of the fifteen study sites. To test the sensitivity of our results to the choice of air pollution metric, we repeated the analysis using distributed lags [21] from 0 to 5 days for each of the air pollutants and excluded the two more distant monitoring sites from this reanalysis.

### Physiologic Measures

#### Blood pressure

The measurement techniques were guided by Campbell et al. [22].After five minutes seated in a quiet environment, six measurements were taken, at one minute intervals, on the right arm using an automated blood pressure device (BpTRU^™^ BP-300) with a cuff 40%-50% of the arm circumference. The mean of the last five measures were used for analysis [23].

#### Lung function

Trained personnel adhered to the guidelines of the American Thoracic Society/European Respiratory Society Task Force quality criteria report for test performance using a KoKo Spirometer ^™^ (Ferraris CardioRespiratory, Pulmonary Data Services, Inc. Louiseville, CO 80027 USA) [24]. Calibration with a three-litre syringe was done prior to each testing session. Subjects were excluded if they couldn’t perform the required test maneuver, had an acute respiratory tract infection, or were more than 24 weeks pregnant. Variables used in analysis were the largest 1-second forced expiratory volume (FEV_l_) and forced vital capacity (FVC) obtained by a minimum of three and a maximum of eight trials [23]. These variables reflect airway diameter and lung volume respectively, and the ratio FEV_1_/FVC reflects airway diameter adjusted for lung volume.

### Statistical Analysis

Lung function was expressed as a percentage of predicted based on age, height, and gender using previously developed prediction equations, with 100% percent being the normal average point estimate [25]. To determine if mental health influences the magnitude of effect of air pollution on mental health we used generalized linear mixed models with sampling weights. Covariates tested for inclusion included age, gender, body mass index, ethnicity (Caucasian versus other), greatest household education, household income, active smoking status (daily smoker, occasional, non-smoker), household exposure to passive smoking (daily or almost daily, other), frequency of alcohol consumption (less than 1, 1,2–3,4–6,7 days weekly), mean daily temperature and relative humidity. All main effects and first order interaction products were considered. If The Wald Chi-Square statistic p value was less than 0.10 for a main effect or interaction product, it was retained. The final model contained the selected variables and also covariates which were significant at p <0.05 or if they confounded the exposure-outcome relationship (i.e. a change of 10% in the coefficient for exposure). The final model included age, gender, greatest household education, household income, smoking (daily smoker, occasional and non-smoker), and the temperature. Relative humidity and barometric pressure were not significant. Data management and regression modeling were completed in SAS, v9.1 (Cary, NC, USA). We calculated and presented the percentage change in physiologic measure for an interquartile range increase in air pollutant. Finally, to determine if mental health influences the magnitude of effect of air pollution on physiology, we t-tested the differences between the effect sizes in the categories of mental health.

## Results

Air pollution concentrations were relatively low and well within the Canadian and U.S. National Ambient Air Quality Standards [26]. (Table 1). Of the 1,883 participants between the ages of 6 and seventeen years old, 88% were reported to be happy vs. other, six percent had a reported mood disorder, and 85% reported no problems (a score of zero) on the SDQ (Table 2). The children were predominantly Caucasian and from households with education beyond secondary school, and a middle class income. Lung function, heart rate and blood pressure were unremarkable.

**Table 1:** Ambient air pollution concentrations during the 353 days of testing measured at the stationary monitors nearest to the study sites between 2007 and 2009.

	Minimum	Maximum	Mean	Standard Deviation	Interquartile range
**Relative Humidity (%)**	36.58	99.21	72.93	12.72	17.79
**Temperature (°Celsius)**	-12.66	27.17	8.05	8.83	14.86
**NO**_**2**_ **(24-hr mean, μg/m**^**3**^**)**	0.88	40	11.74	7.92	11.48
**PM**_**2.5**_ **(24-hr mean, μg/m**^**3**^**)**	0	38.88	5.60	4.52	3.96
**O**_**3**_ **(8-hr max, ppb)**	2	83	29.50	11.80	17

**Table 2:** Sociodemographic and physiologic indicators by mental health status indicators among children 6 to 17 years old who participated in the Canadian Health Measures Survery, 2007–2009. Results are means (95% CI) unless otherwise specified as percentages.

	Happiness		Mood Disorder		SDQ—emotional symptoms	
	Happy n = 1658	Other n = 225	Absent n = 1693	Present n = 190	No Symptoms n = 1602	Some Symptoms n = 281
**Subject Characteristic**						
**Sociodemographics**						
Age, yr n = 1883	10.9 (10.8, 11.1)	12.6 (12.2, 13.0)	11.1 (10.9, 11.3)	11.2 (10.6, 11.8)	11.2 (11.0, 11.3)	10.9 (10.5, 11.3)
Ethnicity, Caucasian, % (n = 1836)	81.0 (80.2, 81.8)	74.7 (74.1, 75.3)	84.0 (83.0, 85.0)	77.9 (77.1, 78.7)	83.4 (83.2, 83.6)	82.6 (82.1, 83.6)
Highest household education, greater than high school, % (n = 1836)	84.2 (83.1, 85.3)	77.1 (74.5, 79.7)	98.0 (96.7, 99.3)	54.7 (53.1, 56.3)	88.4 (88.2, 88.5)	80.66 (80.2, 88.5)
Total household income, $10^3^ (n = 1395)	91.1 (87.4, 94.9)	77.8 (70.2, 85.4)	92.0 (79.4, 104.6)	89.5 (85.9, 93.0)	92.5 (88.7, 96.4)	74.0 (67.3, 80.7)
Smokers, daily or occasionally % (n = 256)	15.7 (15.3, 16.1)	27.0 (25.8, 28.2)	12.0 (11.5, 12.5)	19.5 (18.7, 20.3)	13.5 (13.1, 13.9)	14.2 (13.9, 14.5)
**Lung Function (n = 1711)**						
FEV_1_^(% predicted)^	99.3 (98.7, 99.9)	99.7 (98.1, 101.5)	99.1 (11.0, 11.3)	99.2 (96.8, 101.5)	99.4 (98.7, 0.1)	99.4 (97.8, 101.0)
FVC (% predicted)	102.3 (101.7, 102.9)	103.1 (101.5, 104.8)	102.4 (101.8, 102.9)	102.3 (99.8, 104.8)	102.3 (101.7, 103.0)	102.8 (101.1, 104.4)
FEV_1_/FVC (%)	97.2 (96.9, 97.6)	96.9 (95.8, 98.1)	97.2 (96.8, 97.6)	97.0 (95.7, 98.4)	97.2 (96.8, 97.6)	97.0 (96.0, 98)
**Cardiovascular Function** (n = 1875)						
Heart rate, per minute	77.4 (76.8, 77.9)	76.5 (75.1, 77.9)	77.2 (76.7, 77.7)	77.9 (75.5, 80.2)	77.1 (76.5, 77.6)	79.9 (78.6, 81.2)
Systolic Blood Pressure, mmHg	95.1 (94.7, 95.5)	96.2 (95.0, 97.3)	95.2 (94.8, 95.6)	96.4 (94.6, 98.2)	94.7 (94.3, 95.12)	95.7 (94.6, 96.7)
Diastolic Blood Pressure, mmHg	61.1 (60.8, 61.5)	61.1 (60.3, 62.0)	61.1 (60.8, 61.5)	61.1 (59.8, 62.5)	60.9 (60.5, 61.3)	61.1 (60.4, 61.9)

Compared to others in the study, those who were less happy, had mood disorders, and scored higher on the SDQ were less likely to be Caucasian, more likely to come from households with lower education and income, and were more likely to smoke (Table 2). Physiologic measures (Table 3) and air pollution concentrations (Table 3) did not appear to be related to mental health indicators, with the exception that ozone concentrations were 2 ppb greater in those with some vs. no SDQ symptoms.

**Table 3:** Mean (95% CI) air pollution concentrations measured at the nearest stationary monitoring site on the day of testing stratified by indicators of mental health status among children aged 6 to 17 years old who participated in the Canadian Health Measures Survey, 2007–2009.

	Air Pollutant (mean, 95%CI)		
	NO_2_ (24-hr mean, ppb)	O_3_ (8-hr max, ppb)	PM _2.5_ (24-hr mean, μg/m^3^)
**Emotional Health Indicators**			
**Happiness**			
Happy n = 1658	11.5 (11.1, 11.9)	29.3 (28.7, 29.9)	5.6 (5.4, 5.8)
Other n = 225	12.5 (11.4, 13.6)	29.6 (27.9, 31.3)	6.0 (5.5, 6.3)
**Mood Disorder**			
Absent (n = 1770)	11.3 (9.8, 12.7)	29.0 (26.8, 31.3)	5.6 (4.8, 6.4)
Present (n = 109)	11.7 (11.3,12.1)	29.4 (28.8, 29.9)	5.6 (5.4, 5.8)
**SDQ emotional symptoms**			
No symptoms (n = 1602)	11.7 (11.3, 12.1)	29.0 (28.4, 29.6)	5.6 (5.4, 5.9)
Some symptoms (n = 281)	11.2 (10.3, 12.1)	31.4 (30.0, 32.8)	5.5 (4.9, 6.1)

Differences in results between those who were happy vs. other, were assessed by presenting the association between air pollution and physiology stratified by happy vs. other (Table 4). The observed effects of ozone on heart rate and diastolic blood pressure were significant in those who did not report being happy, but were not significant in those who reported being happy, despite the much greater sample size in the latter group. Diastolic blood pressure was significantly greater in association with PM_2.5_, but not among those who reported being happy. The ozone- FEV_l_ /FVC, and the PM_2.5_– FEV_l_ associations were only significant in the group that did not report being happy. Although the point estimates of the observed effects of NO_2_ on heart rate and blood pressure were greater in the group that did not report being happy, none of these associations reached statistical significance.

**Table 4:** The percent change in heart rate, blood pressure, and lung function for an interquartile range increase in air pollutant concentration stratified by happy v. other among children aged 6 to 17 years old who participated in the Canadian Health Measures Survey, 2007–2009 ^a^.

Physiologic Outcome	Degree of Happiness	NO_2_ (IQR = 11.48)	O_3_ (IQR = 17.38)	PM_2.5_ (IQR = 3.96)
Heart Rate (bpm)	Happy	0.16 (-0.74, 1.05)	0.16 (-0.74, 1.05)	0.04 (-0.47, 0.54)
	Other	1.15 (-1.29, 3.59)	**3.1 (0.42, 5.65)** ^b^ *	0.48 (-1.04, 1.99)
Systolic Pressure (mmHg)	Happy	0.55 (-0.1, 1.2)	0.55 (-0.1, 1.2)	0.03 (-0.35, 0.4)
	Other	0.68 (-1.14, 2.5)	2.6 (0.72, 4.48)*	0.50 (-0.65, 1.65)
Diastolic Pressure (mmHg)	Happy	0.44 (-0.18, 1.05)	0.44 (-0.18, 1.05)	**0.99 (0.33, 1.65)**
	Other	0.64 (-0.75, 2.03)	0.64 (-0.75, 2.03)	0.54 (-0.35, 1.43)
FEV_l_ (%)	Happy	-0.66 (-1.77, 0.45)	-0.66 (-1.77, 0.45)	-0.42 (-1.05, 0.21)
	Other	-0.98 (-3.8, 1.84)	-0.98 (-3.8, 1.84)	**-1.68 (-2.15, -0.21)**
FVC (%)	Happy	-0.23 (-1.32, 0.87)	-0.23 (-1.32, 0.87)	-0.51 (-1.13, 0.12)
	Other	-1.1 (-3.98, 1.77)	-1.1 (-3.98, 1.77)	-0.57 (-2.6, 1.46)
FEV_l_ /FVC (%)	Happy	-0.36 (-1, 0.28)	-0.36 (-1.00, 0.28)	0.06 (-0.31, 0.42)
	Other	-0.45 (-2.4, 1.51)	-2.15 (-3.62, -0.68)*	-0.3 (-1.63, 1.02)

^a^ Results are adjusted for age, gender, education, income, active and passive smoking (active, passive) and alcohol use

^b^ Bold font indicates the 95% confidence intervals exclude zero.

*Interaction term is significant at p ≤ 0.05 between those are happy vs. other.

Differences in findings between those with and without SDQ symptoms were examined by presenting the association between air pollution and physiology stratified by the presence or absence of more SDQ symptoms (Table 5). Results were most consistent for associations with ozone. Compared to those scoring lower on the SDQ, those scoring higher had greater and statistically significant increases in heart rate, systolic and diastolic blood pressure, and significant decreases in FEV_l_ and FEV_l_ /FVC. No significant ozone-related changes were seen in those with lower SDQ scores despite this group having a larger sample size. Increased exposure to PM_2.5_was associated with an increase in heart rate and decrease in FEV_l_, but only in those with SDQ symptoms. No significant associations were seen with NO_2_. However, compared to those with an SDQ score of 0, among those with higher scores, point estimates of heart rate and blood pressure were higher and lung function lower.

**Table 5:** The percent change in heart rate, blood pressure, and lung function for an interquartile range increase in air pollutant concentration stratified by the number of emotional symptoms among children aged 6 to 17 years old who participated in the Canadian Health Measures Survey, 2007–2009 ^a^.

Physiologic outcome	SDQ Emotional Symtoms	NO_2_ (IQR = 11.48)	O_3_ (IQR = 17.38)	PM_2.5_ (IQR = 3.96)
Heart Rate (bpm)	None	0.07 (-2.17, 2.37)	3.37 (-3.28, 10.02)	2.02 (-2.88, 7.18)
	Some	2.49 (-1.36, 6.5)	**6.4** ^b^ * **(1.72, 11.28)**	6.53* **(0.81, 12.56)**
Systolic Pressure (mmHg)	None	0.13 (-1.8, 2.08)	0.61 (-3.55, 4.77)	0.01 (-2.2, 2.21)
	Some	2.08 (-2.4, 6.76)	4.13* **(1.16, 7.10)**	0.63 (-1.84, 3.1)
Diastolic Pressure (mmHg)	None	0.42 (-1.19, 2.05)	0.02 (-4.41, 4.45)	0.04 (-1.31, 1.4)
	Some	1.62 (-0.19, 3.45)	4.67* **(1.19, 8.15)**	0.54 (-0.38, 1.46)
FEV_1_ (%)	None	-0.06 (-1.63, 1.53)	-0.48 (-2.52, 1.56)	-0.12 (-3.46, 3.35)
	Some	-0.55 (-3.27, 2.24)	**-5.97 (-10.57, -1.37)**	-5.57* **(-8.89, -2.13)**
FVC (%)	None	-0.14 (-2.18, 1.94)	-0.2 (-2.2, 1.83)	-0.07 (-2.2, 2.10)
	Some	-0.26 (-1.79, 1.3)	-1.78 (-6.19, 2.83)	-4.77* **(-7.97, -1.46)**
FEV_1_/FVC (%)	None	-0.4 (-1.33, 0.55)	-0.65 (-3.89, 2.6)	-0.01 (-1.48, 1.48)
	Some	-1.53 (-3.34, 0.32)	-5.91* **(-9.91, -1.91)**	1.0 (-1.39, 3.45)

^a^ Results are adjusted for age, gender, education, income, active and passive smoking (active, passive) and alcohol use

^b^ Bold font indicates the 95% confidence intervals exclude zero.

*Interaction term is significant at p ≤ 0.05 between those with and without SDQ symptoms.

The effect of a mood disorder was investigated by presenting the association between air pollution and physiology stratified by the presence or absence of a reported mood disorder (Table 6). Again, similar to reported happiness and the SDQ, results were most consistent for ozone. Compared to those without a mood disorder, those with a mood disorder scoring lower on the SDQ, those scoring higher had greater and statistically significant increases in systolic and diastolic blood pressure, and significant decreases in FEV_l_. No significant ozone-related changes were seen in those without a mood disorder except for FEV_l_ /FVC. However, the point estimate for FEV_l_ /FVC was more negative for those with a mood disorder. The lack of significance was likely due to the almost 20 fold smaller sample size in the group with a mood disorder. Increased exposure to PM_2.5_was associated with a significantly increased systolic blood pressure and a decreased FEV_l_ /FVC but only in those with a mood disorder. The point estimates of the NO_2_ effects on lung function were more negative for those with a mood disorder and significant for FVC and FEV_l_ /FVC.

**Table 6:** The percent change in heart rate, blood pressure, and lung function for an interquartile range increase in air pollutant concentration stratified by the presence or absence of a reported mood disorder among children aged 6 to 17 years old who participated in the Canadian Health Measures Survey, 2007–2009 ^a^.

Physiologic outcome	Mood Disorder	NO_2_ (IQR = 11.48)	O_3_ (IQR = 17.00)	PM_2.5_ (IQR = 3.96)
Heart Rate (bpm)	Present	1.8 (-2.51, 6.11)	2.10 (-1.29, 5.50)	0.07 (-2.04, 2.18)
	Absent	0.39 (-0.51, 1.29)	-0.36 (-1.16, 0.44)*	-0.25 (-0.76, 0.25)
Systolic Pressure (mmHg)	Present	1.24 (-1.69, 4.18)	**3.75 (1.62, 5.89)**	**1.94 (0.56, 3.32)**
	Absent	0.06 (-0.56, 0.69)	-0.44 (-1.00, 0.12)*	0.06 (-0.30, 0.42)*
Diastolic Pressure (mmHg)	Present	-0.76 (-3.57, 2.04)	**3.02 (0.86, 5.17)**	-0.9 (-2.27, 0.48)
	Absent	-0.32 (-0.9, 0.25)	-0.2 (-0.72, 0.31)*	-0.02 (-0.35, 0.31)
FEV_1_ (%)	Present	-2.53 (-7.69, 2.63)	**-6.18 (-10.63, -1.73)**	-0.77 (-3.55, 2.01)
	Absent	**-1.95 (-3.06, -0.83)** ^b^	-1.08 (-2.08, -0.07)*	-0.09 (-0.74, 0.55)
FVC (%)	Present	**-5.82 (-11.27, -0.37)**	**-7.62 (-12.31, -2.92)**	-2.86 (-5.79, 0.08)
	Absent	-1.75 (-2.83, -0.67)*	-0.52 (-1.5, 0.47)*	-0.05 (-0.68, 0.58)*
FEV_1_/FVC (%)	Present	**-3.05(-5.8, -0.3)**	-0.91(-3.33, 1.51)	**-2.43 (-3.84, -1.02)**
	Absent	0.2 (-0.45, 0.84)*	**-0.65 (-1.23, -0.07)**	-0.04 (-0.42, 0.33)*

^a^ Results are adjusted for age, gender, education, income, active and passive smoking (active, passive) and alcohol use

^b^ Bold font indicates the 95% confidence intervals exclude zero.

*Interaction term is significant at p ≤ 0.05 between those with and without a mood disorder.

Reanalysis using distributed lag models [21] for each pollutant while excluding the two study sites with monitors greater than 25km away made very little difference to the pattern and statistical significance of the results. One notable change was that an interquartile increase in NO_2_ was associated with a 1.76% (95%CI 0.62, 2.91) increase in systolic blood pressure in those who were less happy, but no significant effect was observed in those who were happy. This additional finding further supports the results using same day air pollution concentrations.

## Discussion

We observed that, in several instances, increased ambient air pollution was associated with higher heart rate, blood pressure, and lower lung function among those with evidence of poorer mental health. Among those with better mental health, no adverse effects of air pollution were observed. Of the three pollutants measured, the most frequent associations were with ozone. Of the three mental health indicators, the most frequent associations were with a health care professional- diagnosed mood disorder.

### Strengths and Limitations of the Study

Using physiologic measures of lung function avoided the possibility of a self-reporting bias whereby unhappy subjects may be more or less prone to report illness than happier subjects. The validity of the SDQ questionnaire has been well demonstrated. Internationally, it is reported to be one of the most frequently used screening instruments for child and adolescent mental health purposes’ [19]. A study of approximately 18,000 British schoolchildren concluded that the SDQ scores offered a relatively accurate and unbiased assessment of the prevalence of mental health disorders compared to the opinion of a clinician [20]. Cross-sectional studies have an inherent weakness in that they take a snapshot in time, in this case the day the subject was studied. A longitudinal study would be required to determine if the observed effects are from relatively acute or chronic exposure. On average, subjects with exposures to greater concentrations of air pollution on the day and week of the study will also be chronically exposed to greater concentrations on a chronic basis than subjects who are less exposed on the day of the study. It would have been advantageous to have an estimate of air pollution at the site of the participant’s residence, but privacy regulations of Statistics Canada did not allow us to have postal code information. Fortunately, the majority of people lived within 11 km of the study site, and monitors were relatively close to the study site with two exceptions. Excluding these exceptions did not materially change the findings. Ambient monitoring, which has been used effectively for decades to detect air pollution health effects is recognised to be an inaccurate measure of personal exposure, especially since people spend the majority of their time indoors. This problem would be expected to bias results towards the null, showing no effect of air pollution on health. Thus, positive findings are likely conservative estimates of the true effect size.

These findings are unlikely to be confounded given that we considered the following covariates: age, gender, body mass index, ethnicity, education, income, active and passive smoking, alcohol consumption, and weather variables. Cross-sectional studies are limited by being a snap short in time, making it difficult to know which event came first. One could argue that mental illness might be precipitated by a medical illness or its treatment. However, the small but significant reductions in lung function and elevations in blood pressure we detected are unlikely to be symptomatic. Even if medicated, anti-hypertensive medications and inhaled medications for airways disease are unlikely to cause a mood disorder.

The findings are unlikely to be due to chance alone given that there were 23 positive associations between different physiologic measures and different air pollutants among those with less favourable mental health indicators, but no significant associations among those with more favourable mental health.

The existence of previous studies with similar to ours would provide evidence for the association being causal, but our study is unique, the first of its kind to our knowledge. However, there have been studies demonstrating associations between adverse health effects, and both air pollution and depression [1, 27–29]. We have shown that air pollution is more likely to have a measureable adverse in those with indicators of poorer mental health. There are plausible mechanisms: self-care behaviours, social and financial support, symptom reporting, and systemic inflammation. Based on a systematic review of the literature between 1968 and 1998, DiMatteo et al. [9] calculated an odds ratio of 3.03 (95% CI 1.96–4.89) between depression and treatment noncompliance. Depression has been reported to adversely affect employment and social functioning, and a fear of discrimination may possible impede accessing care [10]. In the present study, however, results were adjusted for both income and education. A cross-sectional population study from Alberta, Canada revealed increased symptom reporting in those who scored higher on a standardized psychiatric symptom questionnaire, the Ilfeld Psychiatric Symptom Index after adjusting for level of lung function among subjects who had never smoked and had no diagnosed chronic medical illnesses [30]. Kiecolt-Glaser and Glaser [7] presented an argument that psychological stress and depression increase IL-6, a marker of inflammation, and decrease cellular and humoral immunity. Among 3024 subjects between 70 and 79 years of age, those who scored higher on a standardized depression scale also had significantly higher levels of IL-6, TNF, and CRP [8].

Our findings are important for several reasons. It is recognized that pre-esixting physical illness increases susceptibility to the adverse effects of air pollution. Now we have evidence that mental illness may confer a similar vulnerability. There are practical reasons to identify susceptible subgroups. Setting air pollution guidelines based on observed effects in the general population may be insufficient to protect especially vulnerable subgroups. Also the most susceptible may derive the most benefit from reduction in air pollution. Based on modelling the expected health benefits of reducing emissions from fossil-fuel power plants, Levy et al. [3] reported disproportionate mortality reductions among African Americans, those with less than a high school education, and those with diabetes. Identifying the especially vulnerable would be a first step in focusing prevention strategies. For example, the Air Quality Health Index communicates to the public both a summary of the air quality and preventative advice which differs between those with and without an elevated risk due to chronic lung or heart disease and the general population [2]. If mental illness identifies a subgroup at higher risk of adverse health effects from air pollution than the general population, knowing this may help explain variability between studies with different proportions of mental disorders. The magnitude of the observed effect of air pollution in any given study will be influenced by the proportion of vulnerable people exposed [1].

## Conclusions

Our results suggest that, among children, mental health may influence susceptibility to the adverse physiologic effects of air pollution. We recommend confirmatory studies, including longitudinal studies, and testing whether or not mental disorders modify the effect of air pollution on other physical health outcomes.

## Abbreviations

CHMS: Canadian Health Measures Survey
FEV_l_: 1-second forced expiratory volume
FVC: forced vital capacity
HS: a self-reported happiness scale
NO_2_: nitrogen dioxide
O_3_: ozone
PM _2.5_: particulate matter of median aerodynamic diameter less than 2.5 microns
SDQ-ES: the Emotional Symptom Subscale of the Strengths and Difficulties Questionnaire
